# P-272. Prevalence and Predictors of Multi-drug Resistant Organisms in Critically Ill Patients with Opioid Use Disorder and Infection: A Multicenter Retrospective Cohort Study

**DOI:** 10.1093/ofid/ofae631.476

**Published:** 2025-01-29

**Authors:** M Zeeshan Rizwan, Ryan W W Stevens, Haris Akhtar, Julie Cunningham, Kristin Cole, ognjen Gajic

**Affiliations:** Mayo Clinic, Rochester, Minnesota; Mayo Clinic, Rochester, Minnesota; Mayo Clinic Rochester, Rochester, Minnesota; Mayo Clinic, Rochester, Minnesota; Mayo Clinic, Rochester, Minnesota; Mayo, Rochester, Minnesota

## Abstract

**Background:**

The opioid crisis and the increasing prevalence of multi-drug resistant organism (MDRO) infections pose a threat to public health. Opioid Use Disorder (OUD) patient are prone to MDRO colonization and infections, thus potentially leading to adverse outcomes during periods of critical illness. Understanding the prevalence and predictors of MDRO infections within this vulnerable population is critical to improving patient outcomes by optimizing interventions and treatment plans.

**Figure:**
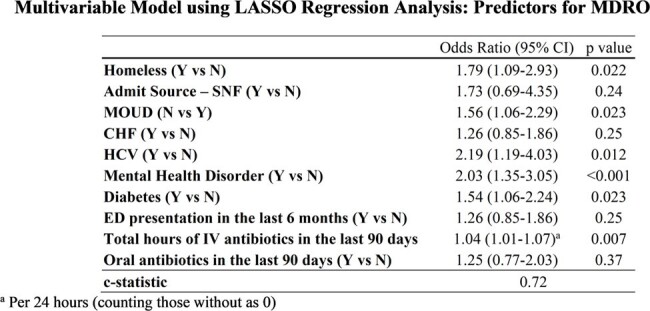

**Methods:**

We conducted a retrospective, multicenter, cohort study to evaluate the prevalence of MDRO isolation among adults with OUD admitted to an intensive care unit (ICU) with an infection across four Mayo Clinic regions. Patients were classified to the MDRO cohort if an MDRO was isolated from standard cultures or rapid diagnostic testing within 48 hours of admission. We utilized descriptive statistics, univariate and multivariate least absolute shrinkage and selection operator (LASSO) regression analysis including random effect to account for site variability, to analyze demographic data, clinical characteristics, and outcomes.

**Figure:**
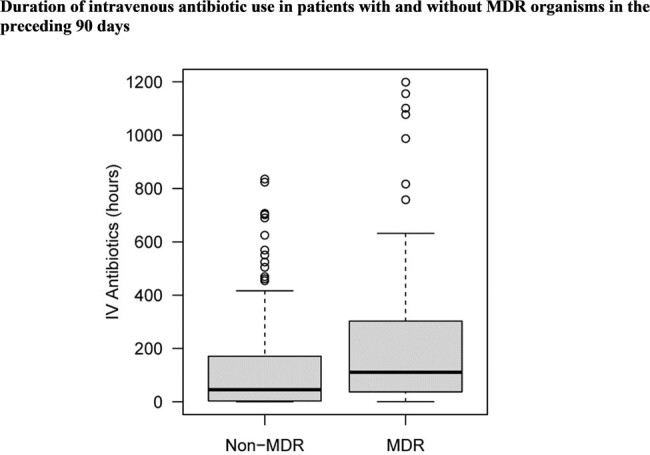

**Results:**

790 critically ill adult OUD patients with infection-related admission were included in the study cohort. The prevalence of MDRO isolation was 22.5% (n=178), with MRSA being the predominant isolated MDRO. In LASSO regression analyses, we identified homelessness (OR: 1.79, p = 0.022), no Medications for OUD (MOUD) treatment (OR: 1.56, p=0.023), and positive Hepatitis C Virus (HCV) status (OR: 2.19, p = 0.012) as significant predictors for isolation of an MDRO. Intravenous (IV) antibiotic use in the preceding 90 days was also positively associated (OR: 1.04 per 24 hours, p = 0.007), alongside mental health disorders (OR: 2.03, p < 0.001) and diabetes (OR: 1.54, p = 0.023).

**Figure:**
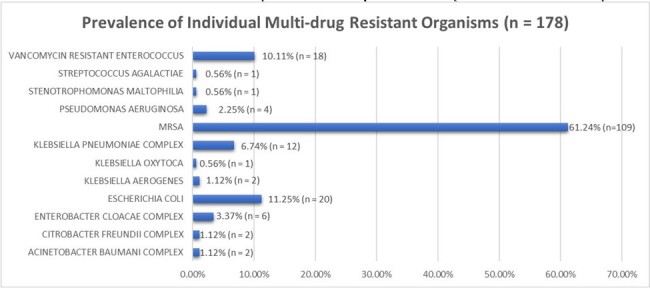

**Conclusion:**

The high prevalence of MDRO isolation in critically ill OUD patients with infection is associated with complex social and clinical factors.

**Disclosures:**

**All Authors**: No reported disclosures

